# Gaze behavior reveals automaticity and attention allocation during music teaching vs. observing

**DOI:** 10.16910/jemr.17.2.3

**Published:** 2024-07-22

**Authors:** Robin S. Heinsen

**Affiliations:** Miami University Oxford, OH, USA

**Keywords:** Eye movement, mobile eye tracking, gaze, attention, music, expertise, automaticity

## Abstract

In a unique case-study approach in which I served as both the research participant and the experimenter, I wore eye-tracking glasses while teaching a brief music lesson to two university students learning trumpet, then approximately two weeks later, I watched a video of the lesson and tracked my gaze again. To investigate unconscious perceptual processes engaged during music teaching, I compared my attention allocation while teaching to my attention allocation during selfobservation.

My gaze behavior while teaching revealed a high level of automaticity regarding lesson sequencing and allocation of attention. Strategic moment-to-moment shifts in attention between the two students occurred entirely below my conscious awareness, yet post hoc analyses revealed precisely timed changes that were related to momentary goals. While watching the video, absent the demands of behavioral interaction and momentary decision-making, I directed more sustained attention to both students than I had while teaching.

These results reveal important features of “teacher thinking” that are not directly observable or typically construed as conscious behavior. That this component of teaching practice does not involve volitional control suggests that teachers’ descriptions of their thinking may not reveal to novices important elements of pedagogical expertise.

## Introduction

Eye-tracking research in music contexts has examined music reading (47; 28; 55; 62; 63; 65; 79), music performance and hand/eye coordination (11; 56), music performance and communicative gaze (75), and the effects of music stimuli on visual processing (29; 37), but research on eye movement and music teaching is in an earlier phase of development (e.g., 41; 42; 57; 72). Studying the gaze behavior of music teachers requires deep understandings of music as dynamic, interactive, and refined over time, and of skillful teachers as effective shapers of learner behavior through the creation of experiences that move learners increasingly closer to instructional goals. In music classrooms, students’ skill development is evident throughout the course of a lesson, and music teachers can assess momentary progress and make quick decisions that affect performance outcomes. Expert music teachers notice aspects of their students’ behavior and performance that may escape the notice of less expert teachers, addressing potential problems quickly and thoroughly, and making substantive changes in the way their students perform (21). These interactions allow for detailed observations of teacher attention allocation in relation to student behavior and music performance.

Visual information is particularly useful in formulating action plans and guiding behavior, so much so that visual focus has been shown to be a reliable indicator of cognitive attention, even when integrated with auditory stimuli (8; 16). When individuals orient to new environments, prepare to take action, and solve problems, they engage multiple cognitive processes—attending to available options, evaluating the most likely choices, choosing and then verifying the selection—and these processes are reflected in eye movements when individuals look at the items they think about (32; 38). Visual targets that become associated with highly rewarding outcomes capture attention more so than do targets with lesser predicted reward (53), and individuals repeatedly attend to stimuli that have proven useful in the past. Thus, “attentional biases” are in effect a prioritization of perceptual targets that facilitate the accomplishment of rewarding, meaningful outcomes (1), and as such are learned behaviors. Decisions about where to focus visual attention are influenced by combinations of features in the immediate environment and past experiences stored in memory. As individuals fixate and re-fixate in ways that are advantageous, these repeated attentional patterns become increasingly automatized (1). Although some of this activity is consciously controlled, much of it operates below conscious awareness.

As understanding deepens and skills develop, conscious attention shifts to increasing levels of abstraction or construal (64; 73), a process that is facilitated by access to a rich store of retrievable memories that enable recognition of meaningful patterns and formulation of actionable predictions. Experts’ knowledge and accumulated experience affects their perception, attention, and decision making, and this is often encapsulated in broad models like situation awareness (23). An ongoing challenge in studying expertise is developing protocols that illuminate experts’ thinking when so much of their thought processes are automatized and unconsciously controlled (43; 45; 44). Individuals are generally unreliable narrators of their own thinking (15; 61; 70), and most participants verbalize only a small portion of their actual thinking and decision making. Guan et al. (33) attributed such differences to the different levels of abstraction and data density that occur in verbalizations versus what can be captured in eye movements. Because expert gaze behavior is a manifestation of expert thinking, (51), eye movements provide insight into automatized cognitive processes and give greater detail than may be obtained through interviews and other forms of self-report (25).

Many features of teacher expertise (for a comprehensive list, see 6, 7) become discernible by using eye tracking to investigate gaze patterns that are indicative of cognitive processing, automaticity, and attention allocation in skillful teachers (e.g., 49). Eye tracking can offer insight into how and when skillful teachers recognize student behavior patterns, which subtle cues or elements of the classroom environment they are most attentive to, and how they distribute attention and monitor their classroom (50). Eye tracking technology enables researchers to study gaze patterns of expert and novice teachers while watching videos and actively teaching, and many studies have focused on findings involving attention distribution among students, classroom monitoring and scanning behaviors, and sensemaking strategies (5).

Expert teachers fixate informative targets in pursuit of goals based on the recognition of patterns of student behavior that have been stored in memory over years of experience. Wolff et al. (76) found that when expert teachers observed recordings of classroom episodes, they tended to fixate and re-fixate parts of students’ bodies that may provide nonverbal indicators of attention and emotion (e.g., shoulders, arms, chest, elbows, and hands). Both van den Bogert et al. (74) and Wolff et al. (76) found that when observing a classroom disruption, expert teachers fixated other students seated close to the commotion rather than the disruptive student, reflecting expert teachers’ understanding of how classroom disruptions evolve and leading to their skillful monitoring of informative or predictive targets (e.g., students who are at risk of being distracted).

Tracking gaze during live teaching affords additional insight during momentary interactions between teachers and students and increases ecological validity (52). Teacher gaze patterns change depending on the instructional task (34), suggesting that different thought processes underlie each component of a lesson. Expert teachers’ thought processes are well practiced and automatized, and experts tend to fixate a greater number of students and follow up on instructional directives more often than do novices (14; 58), especially with students whose cognitive ability or behavior regulation is lower than average. Teacher eye contact has also shown repeatedly to be an integral component of classroom management and shaping student behavior (e.g., 35; 36; 40; 59; 78), as teachers strategically use eye contact and gaze cueing (22) to direct students’ attention and to keep students focused on the class activity. However, because so many variables are present in dynamic classrooms, various studies related to classroom teaching and eye tracking resulted in the authors obtaining inconclusive or difficult-to-interpret results (e.g., 69; 77), underscoring the importance of study design when examining teacher expertise.

Music classrooms are inherently different from classroom settings that have been previously researched using eye-tracking technology, in large part due to ongoing opportunities for music teachers to assess overt indicators of student learning by their accomplishment of performance goals. Brief segments of instructional time that focus on identifiable music performance goals, which Duke (17, 18) calls rehearsal frames, makes possible the identification of momentary relationships between what teachers do and what students accomplish. Using the rehearsal frame as a unit of analysis for teaching is advantageous in that it reveals relationships among teacher and student behaviors that contribute to the accomplishment of proximal and distal goals (e.g., 12; 13; 19; 57; 68).

There are consistent differences between expert and novice music teacher gaze behavior that mirror findings in other areas of expertise (e.g., 31; 54; 66): expert music teachers tend to fixate longer on targets that are relevant to the accomplishment of performance goals (e.g., a flute player’s embouchure or a violinist’s bow alignment) and are likely to ignore salient but unimportant aspects of the environment. These findings have remained consistent across multiple musical contexts, including watching videos of student performers (42), teaching individual and group lessons ( 39; 57), observing teaching (4), coaching chamber ensembles (39), and reading large ensemble scores (41).

Specifically studying the ways expert music teachers allocate attention during instruction can bring new information to the study of expertise in teaching by providing greater insight into how teachers set proximal goals and make momentary decisions that drive instruction, extending beyond what self-reports, think-alouds, or systematic behavioral analyses can reveal (25; 45; 48). Little is known about how music teachers divide attention among multiple students, how music teachers make decisions about what to do next when monitoring the progress of multiple performers, or even how attention functions differently when observing videos compared to teaching live. The explicit momentary measurement and analysis that eye-tracking methodology affords is key to furthering our understanding of how expert music teachers conceptualize their domain and effect change in learners.

### Purpose

The purpose of the present study was to document my own allocation of attention while teaching a brief group music lesson to uncover evidence of automaticity and unconscious thought processes that drive my teaching behaviors. This is a case study and pilot study, but it is autoethnographic in the sense that I collected data on myself. It is a somewhat proof-of-concept project that is intended to provide baseline data regarding momentary attention allocation of a skillful teacher and prompt further work in this field of research. I recorded my gaze first while teaching, and again while watching a recording of the same lesson several weeks later. This study compares my gaze behavior while teaching a lesson to my gaze behavior while observing that same lesson, and it also analyzes in extreme detail how I pursued the accomplishment of a single musical goal in a single rehearsal frame, as informed by my eye movements.

This study is one of the first moment-to-moment analyses of a representative unit of music teaching in a dynamic environment, thus expanding the findings of Marcum (57), Hicken and Duke (42), and Haataja et al. (34). There is a tremendous amount to be learned from deeply analyzing small episodes of teacher-learner musical interactions, but no previous research has explored this territory with enough detail or technological facility to fully understand the attentional mechanisms that underlie expert music teaching in context, and there are no data about momentary allocation of attention in music teaching when multiple students are present—so collecting data on myself is a reasonable place to start.

I examine my gaze data and interpret my recorded teaching behaviors through the lens of my insight as both a music teacher educator and a researcher of attention allocation to differentiate between which aspects of my attention were consciously controlled, and which were a result of automatic and unconscious processing. When expert teachers, or experts in any domain, are asked to explain what we do when we teach, we often omit important aspects of thinking and behavior that have become automatized or subsumed into larger abstractions of events. When experts talk to novices about teaching, or explain our decision-making, we tend to address only conscious attention because we are often unaware of this unconscious monitoring.

The setting of a small group music lesson, and the clarity of the lesson task, limit the number of potential attentional foci and simplify the context enough to draw clear conclusions about what is capturing my attention and the effect of my actions on momentary student performance. The within-subject model, and the same lesson being used in both conditions, facilitates direct comparisons of attention allocation when teaching a lesson vs. observing a lesson because both the participant and the lesson events are identical.

## Methods

I taught a brief (~5 min) trumpet lesson to two university music students who at the time of the study were enrolled in my brass methods course and learning to play the trumpet. I wore eye-tracking glasses that recorded my gaze during the lesson. I also recorded the lesson from a stationary video camera positioned over my right shoulder and trained on the students, thus approximating my own visual perspective while teaching. Approximately two weeks later, I watched the stationary video of the lesson while tracking my gaze again.

### Participant

My teaching, like that of all experienced teachers, is a result of years of practice allocating attention in ways that facilitate successful student outcomes. I am considered an expert teacher by traditional criteria: I have a terminal degree in music education; my students consistently perform at the top of their level at performance evaluations and competitions; I receive consistent superior performance evaluations and teaching evaluations; I am consistently called upon as a consultant and evaluator; I supervise student teachers; I have over a decade of experience teaching students at multiple age groups and levels of proficiency; and qualitative observations of my teaching have consistently met criteria set forth by Berliner (6, 7) and Duke and Simmons (21).

The two student performers in the lesson, “Charles” and “Allen,” agreed in writing to take part in the lesson and appear on camera for the purposes of the study, but they are not participants in the study itself. After consulting with the Institutional Review Board on the campus of my institution, I was advised that this study did not need an IRB review because it is considered an autoethnography.

### Procedure

Before the lesson began, I fitted myself with Pupil Labs Core eye-tracking glasses and calibrated the equipment using a single-point calibration system. I remained seated to teach the lesson to minimize head and body movement and maintain a more consistent calibration throughout the recording (60). The two students sat approximately 6 feet in front of me and 4 feet apart; there were no music stands. At the time of the recording, the students had been playing trumpet for about 6 weeks.

Approximately two weeks after teaching the lesson, I tracked my gaze again while watching the lesson video as it had been recorded from the stationary camera. I fitted myself with the Pupil Labs glasses, completed the calibration on my 13” MacBook laptop screen, and watched the video on my laptop while listening to the audio through earbuds.

### Lesson events

The activities during the lesson were similar to those practiced during meetings of the full methods class and were familiar to both students. The majority of the 5-min lesson was devoted to buzzing pitches on mouthpieces and echoing 2-pitch melodic patterns, and the lesson culminated with a performance of a melody (Breathin’ Easy from *The Habits of Musicianship*; 20).

My primary goal during the lesson was for the students to play with a clear and steady tone in a series of tasks that increased in technical complexity as the lesson progressed. Instructional time was spent performing sequences of successive approximations leading up to the final performance of the melody. I chose a melody for which the performance goal would be achievable for both students at the end of the 5-min teaching episode. Because the students had been members of my class for six weeks, I knew how each student tended to play and what their capabilities were. Throughout the lesson, Charles struggled with maintaining a clear tone, whereas Allen performed more successfully, which was consistent with their typical performance in class. The students were both highly motivated and attentive, and their social behavior was consistently appropriate and not a conscious concern of mine during planning or during the lesson.

The lesson lasted 4 min 36 sec and contained five rehearsal frames, indicating the pursuit of five proximal goals. We started the lesson on mouthpieces, and at the beginning of the lesson Charles produced an unstable and airy buzz on a concert F (all pitch names hereafter refer to concert pitch), a common problem for him as well as for typical beginning trumpet players. I initiated a sequence of short performance trials that led both students through a series of glissandos (pitch bends) down to Bb and up to F to bring Charles to a properly functioning embouchure (mouth shape and lip movements), which he achieved at the end of the first rehearsal frame. The goal of the second rehearsal frame was to play an F on the full instrument with a clean attack and clear and steady tone. The third and fourth frames focused on 2-note melodic patterns using F and Eb: the third frame consisted of patterns beginning on F, and patterns in the fourth frame all began on Eb. The goal of the fifth frame was to perform the first phrase of *Breathin’ Easy*.

### Data analysis

I used Pupil Labs Player software to analyze my gaze behavior in both the teaching and observing conditions. This software merges information from the eye and scene cameras to create a video of eye movements and scan paths overlaid on the scene (in this case, the two students) using a dispersion-threshold algorithm (67). I used QuickTime and iMovie software to synchronize audio from the stationary camera recording with the Pupil Labs videos. The duration parameters for the software fixation detector were set to 100 ms – 4000 ms (46). To accommodate different amounts of head movement and vestibular ocular reflex (VOR) when teaching versus observing, I analyzed frame-by-frame excerpts of each recording to note the beginnings and endings of fixations, then compared my evaluations to various dispersion threshold settings in the fixation detection algorithm. I selected a 1° dispersion threshold for the observing video and a 2° threshold for the teaching video because those parameters most accurately captured fixation boundaries in relation to head movement and eye movement in each setting (3; 10, 71).

Throughout the lesson, 99% of all my fixations were on parts of the two students’ bodies that were related to playing the trumpet (embouchure/face, body position, breathing apparatus). I did not fixate visual targets that were irrelevant for the accomplishment of proximal goals, which is consistent with other studies of gaze and natural goal-oriented behavior (38) and an indicator of domain expertise (31).

To determine how I allocated attention *between* the two students, given that one was more successful than the other as we progressed through the tasks of the lesson, I defined each student as an area of interest (AOI) and determined how long I fixated each AOI before I switched my gaze to the other AOI (the other student). I operationalized this metric as momentary dwell time, or the summed durations of consecutive fixations anywhere on the student that were at or above the 100 ms minimum threshold.

Thus, the data indicate which student was fixated, and given that there were no fixations in either condition that were on neither AOI, this metric of dwell time on each of the two students seems reasonable even considering the inherent spatial differences and viewing conditions between the teaching and watching conditions. To align my fixations with the proximal goals I sought to accomplish during the lesson, I segmented the lesson into rehearsal frames and organized data analysis into sections based on these frames.

## Results

Results revealed both quantitative and qualitative differences in gaze behavior while teaching and while observing the video of the lesson. This analysis revealed more detail in my own teaching than I was previously aware of, demonstrating a level of automaticity that governs the thought processes of skillful teaching. Experience has shaped my implicit knowledge of when I need to look at students who may need my help and attention and when I can look away, but this behavior operates unconsciously—as evidenced by the level of detail I discovered when analyzing my own attention allocation. Teaching and watching a lesson involve different goals, of course. Although I watched a lesson that I had previously taught myself, I had only vague memories of what had happened in the lesson prior to watching it—not only due to the passage of time, but also because of the number of similar lessons I have taught over my career. In the section that follows, I first discuss summative fixation data followed by an in-depth examination of one representative rehearsal frame, in which I compare my allocation of attention while teaching and while watching the lesson.

### Fixation Data

**Figure 1.**
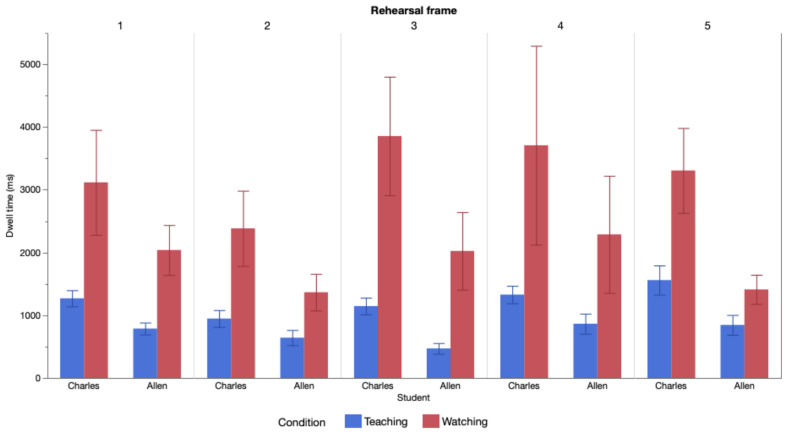
Mean dwell time grouped by rehearsal frame, condition, and student. Note. Bars represent the mean momentary dwell time (summed duration of consecutive fixations on a given student before shifting attention to the other student) in each rehearsal frame; brackets represent standard error. Gaze behavior while teaching (in blue) is characterized by short dwells and more uniform distribution of dwell time than gaze behavior while observing (in red), which is characterized by longer dwells and wide distributions in dwell time. I dwelled consistently longer on Charles than Allen in each condition and each rehearsal frame, but these differences were greater while watching than while teaching.

Details about my gaze behavior across each rehearsal frame are presented in Table 1. Dwell time, the summed duration of consecutive fixations on a given student before shifting attention to the other student, was the most meaningful and reliable representation of divided attention among two individuals in this context because it was less affected by VOR and the dispersion algorithm than were individual fixations, as well as easily standardized across spatial conditions. Mean dwell time grouped by rehearsal frame, condition, and student is displayed in Figure 1.

I analyzed dwell time in a three 3-way ANOVA to assess the effects of condition (Teaching or Watching), student (Charles or Allen), and rehearsal frame (frames 1-5). The main effects of both condition, *F* (1, 280) = 78.47, *p* < .0001, η^2^ = 0.22, and student, *F* (1, 280) = 31.34, *p* < .0001, η^2^ = 0.10, were statistically significant, as was the interaction between condition and student, *F* (1, 280) = 6.79, *p* = .01, η^2^ = .02. This interaction effect is clear in Figure 1. Although I consistently dwelled significantly longer on Charles than Allen in both conditions, and I generally dwelled significantly longer while watching than while teaching, the differences between the two students tended to be greater while watching than while teaching.

Notably, there was no main effect or interactions involving rehearsal frame, and this lack of significant differences demonstrates that my gaze behavior was broadly consistent over the course of the lesson. Performance targets changed from frame to frame, of course, as did the specific tasks the students performed, but the general cadence of how I divided my attention between students did not change. Because the amount of time I attended each student in each condition remained relatively steady, these summative data fall short of illuminating when and why I attended each student. I present below a detailed analysis of the first rehearsal frame to illustrate the amount of data available within a small-scale examination of teaching; detailed analyses of the remaining four rehearsal frames obtained similar results.

**Table 1.**
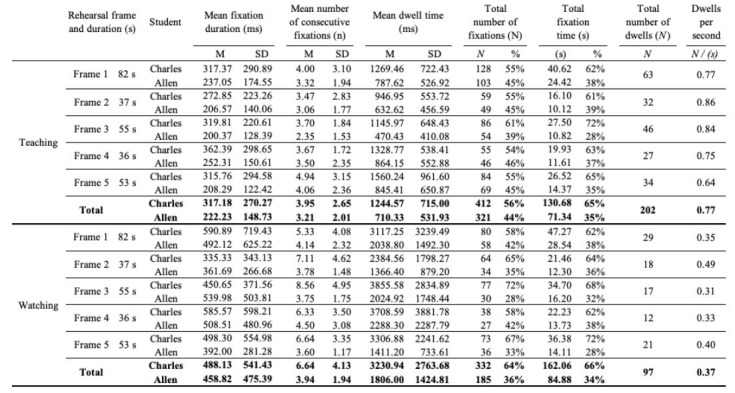
Fixations recorded while teaching and while watching, organized by rehearsal frame.

Note. Consecutive fixations refer to multiple fixations on a single student before shifting attention to the other student, and dwell time is the summed duration of momentary consecutive fixations. Note that fixations while watching tended to be longer and fewer in number than fixations during teaching, and the rate of dwells per second was slower because the fixations and dwells were longer.

### First Rehearsal Frame and Eye Movements While Teaching

The 82-sec rehearsal frame comprised 9 performance trials, and the musical goal was for the students to buzz an F on their mouthpieces with clear and steady tone. Activities included buzzing an F and multiple glissando (pitch bending) patterns between F and Bb.

The description that follows is a momentary analysis of the relationship between gaze data and recorded rehearsal events, not a recollection of my memory of the rehearsal. This analysis would likely look similar if I were interpreting another teacher’s footage, or another teacher interpreted mine (e.g., 34; 39). The added value of analyzing my own data is my acknowledgement of the contrast between my articulable memory of the rehearsal vs. the depth of analysis available with gaze data, suggesting the automaticity of complex thought processes present in skillful teaching that is inaccessible without this technology. Although I taught the lesson myself and believed that I remembered most of what had transpired, my allocation of attention on a scale of milliseconds, which is outlined below, was apparent to me only after I analyzed the recording.

There are several examples of how I pursued the simultaneous goals of helping students play with a better sound on the trumpet (an explicit goal) and keeping students engaged in the activity (an implicit goal). Within each of the 9 performance trials in this frame, I frequently shifted my attention between students while they played. As shown in Table 1 and Figure 1, I prioritized attending to Charles to help him reach the performance goal, evidenced by my longer dwell times, but this did not prevent me from frequently checking in with Allen throughout the lesson, albeit in much shorter durations. I fixated Charles during moments that related to the explicit goal of helping him play with a clear and resonant tone on the trumpet, such as directions for the next trial, glissandos, or attacks, and I mostly fixated Allen during tasks that Charles performed more successfully, such as sustained pitches, releases, or preparatory breaths before an attack, when I could afford to look away from Charles.

My responses to student performance errors demonstrated top-down control of lesson activities. A notable example is captured in Table 2, beginning at 0:30: while I was fixating Allen, Charles played an unsteady sound. I did not immediately move my eyes and shift attention at that moment, but instead gave verbal directions to repeat the performance trial. I then fixated longer on Charles on the next two iterations, yet also afforded a few glances at Allen during moments of the performance trial when I was less likely to obtain useful information from Charles, as mentioned in the previous paragraph, during sustained sounds instead of glissandos. My accumulated experience in this type of context likely inhibited a shift in visual attention away from Allen and onto Charles: I clearly noticed the salient error, as evidenced by my verbal directions and attentional shift on the subsequent performance trial, but because I could control what happened next, I created an opportunity to observe another repetition of the performance task and follow up on Charles’ error instead of reacting suddenly or shifting my gaze immediately. I also continued to attend to both students during the following trial by periodically glancing at Allen while continuing to allocate most of my attention to Charles.

**Table 2.** Timeline comparison of gaze behavior during three performance trials in Rehearsal Frame 1

**Time stamp**	**Event in rehearsal frame**	**Teaching**		**Watching**	
		**Dwell time (ms)**	**Student**	**Dwell time (ms)**	**Student**
0:29	Breathe/cue	1360.8	Charles	6321.01	Charles
0:30	F glissando down to Bb (Charles’s sound is unstable and not on pitch)	1603.48	Allen		
		1058.4	Charles		
		319.2	Allen		
		1239.0	Charles		
		205.8	Allen	5237.4	Allen
0:36	Directions to repeat	966.00	Charles		
0:37	Breathe/cue	940.80	Allen		
0:38	Attack	134.4	Charles		
0:39	F sustain and glissando down to Bb (Charles plays an unexpected sound while holding Bb)	571.06	Allen		
		3309.61	Charles	8341.21	Charles
0:44	“Start on Bb this time (Bb, F, Bb)”	142.80	Allen		
		1012.29	Charles		
0:45	Breathe/cue	474.52	Allen		
0:46	Bb glissando up to F and back down	3213.01	Charles		
		499.8	Allen		
		1180.2	Charles	3343.20	Allen
		327.60	Allen		
0:53	Point to Charles - “Great! Best F so far!”	1688.51	Charles		
0:54	“Try it again, ready….” [breathes]				
		524.89	Allen	2074.85	Charles
		961.80	Charles		
0:55	Bb glissando up to F and back down				
		785.29	Allen	3372.60	Allen
		861.00	Charles		
		1083.48	Allen		
				2276.40	Charles
		470.40	Charles		
		130.2	Allen		
		147.00	Charles		

**Figure 2.**
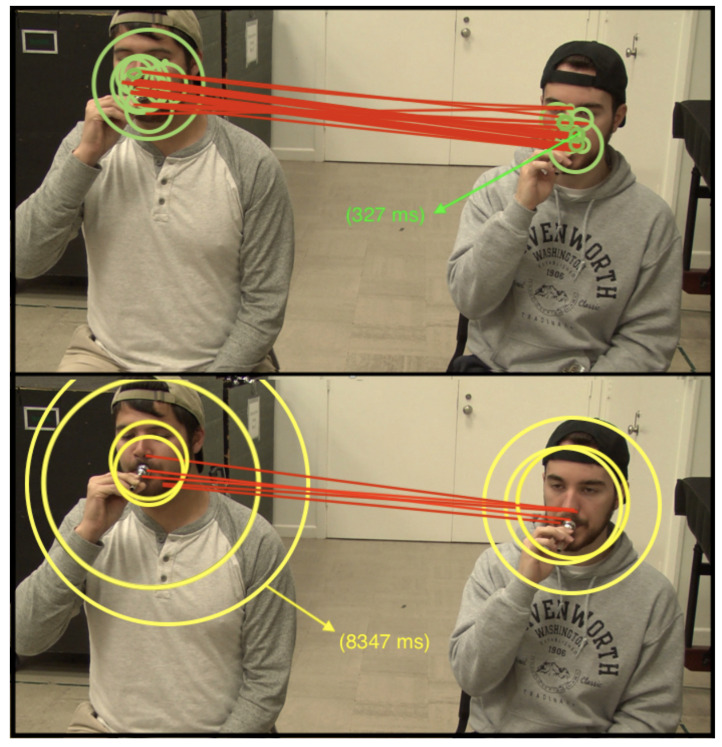
Pictorial representation of gaze behavior comparison Note. This figure represents the same moment in time as Table 2. Each circle signifies one momentary dwell, and one dwell may comprise multiple fixations. The sizes of the circles are proportional to the dwell time. Red lines represent attentional shifts (saccades) from one student to the other. The top image reflects my gaze while teaching, and the bottom image while watching.

There were several additional examples in this frame of strategic attentional control to achieve both musical and behavioral goals, which are outlined below. It is important to reiterate that I only became aware of these moments when I reviewed my gaze footage, supporting the assertion that these cognitive calculations are subsumed into my larger construal of goal-directed teacher-learner interactions. Many fixations were likely determined by what I needed to see from Charles to plan for subsequent performance trials, but it is likely the case that my eye contact with Allen not only allowed me to assess his engagement, but also functioned to reinforce and cue his attention (e.g., 22).

First, as found at 0:46 on Table 2, I fixated Charles during glissandos and Allen on the sustained pitches between glissandos. I budgeted attention toward Charles when he needed it (or when I needed to know something about his playing), and I glanced at Allen during less technically demanding moments when Charles could already perform the task well. Because glissandos are dynamic and require the player to move his embouchure, watching them is more informative to address Charles’ specific performance issue than watching him hold a single pitch.

The second example of strategic attention allocation occurred when Charles performed successfully, after which I directed more visual attention toward Allen on the subsequent trial. This can be seen in 0:53-0:55 of Table 2.

The third type of strategic attentional control relates to social attention and keeping students engaged. During entrance cues or breaths, I either rapidly fixated back and forth between the two students, or I fixated Allen during the breath and then fixated Charles for the attack. These rapid short fixations likely served a dual purpose, both as behavior prompts for the students who receive the attention as well as brief yes/no checks for me to quickly survey the group and determine readiness. These movements are practiced scan paths (2, 30) that have been clearly reinforced and habituated throughout my teaching career, even if their function is as communicative as it is information-seeking.

Fourth, I glanced at Charles’s trumpet, which was in his lap, several times during this rehearsal frame. The students only played their mouthpieces during this frame, and I have no memory of these particular eye movements or fixations. Much like other studies of goal-directed gaze behavior in naturalistic settings (38), perhaps these eye movements reflected that I kept thinking about the trumpet, not just the mouthpiece, and conceivably even reflect my goal of leading Charles eventually to successfully play the trumpet. After the third time I glanced at Charles’ trumpet in his lap, I asked the students to put their mouthpieces into the instrument and we moved on to the next rehearsal frame.

### Eye movements while watching

My gaze behavior while watching the video revealed different patterns of attention than I observed when I was teaching, which can be accounted for by a combination of social attention and prediction mechanisms in addition to the cognitive demands of the task itself. As reflected in Table 2 and Figure 2, while watching the lesson, I generally displayed more sustained attention on each student, exhibiting longer dwell times on each student and switching targets between students at a slower rate. Social attention is immaterial when passively watching a video, of course, and so is the necessity to monitor students’ engagement or their concentration on the lesson. In this different context, I was free to allocate my attention in different ways.

I made more visual responses to errors (reactive saccades) in the watching condition than in the teaching condition, likely because I was not able to control what happened next or predict how many performance trials would occur. Returning to the performance error referenced in Table 2, while watching the lesson I made a reactive saccade to Charles immediately following the salient performance error, then dwelled on him for 8.3 seconds. This greatly contrasts the way I strategically balanced attention between both students while teaching. My behavior as an observer cannot affect the trajectory of a video, leaving me only to react to what is happening in the moment or aim my attention where I predict something may soon be informative.

My attention was also notably different when observing spoken feedback and directives. While watching the video, I repeatedly fixated Allen while Charles was receiving feedback, perhaps gauging his reaction to the information or the performance trial (similar to van den Bogert et al., 74, re: classroom management).

It would be possible to generate a long list of many more examples of these contrasting attentional patterns and strategic attentional control that occurred beyond the first rehearsal frame and over the course of the lesson, but that is not the goal of this study. The most meaningful takeaways are not lists of examples, but the high frequency of their occurrences, the consistency of their patterns, and fact that they governed my attention in ways that I was previously unaware.

## Discussion

This analysis describes my attention allocation while teaching and observing a group music lesson to two students, and the data reveal markedly different attentional patterns and thought processes when I teach compared to when I observe. This study compares two conditions of the same person’s gaze during the same lesson, but the differences in gaze behavior are stark because the thought processes that underlie each experience are drastically different. I interact with preservice teachers daily, and this study challenged me not only to realize I was omitting information about my own thinking when I communicate with them, but also to reconsider the value for preservice teachers of observing others teach.

This analysis revealed to me the extent of my own unconscious attention allocation while teaching. Despite my experience with both eye tracking and teaching methodology, and my understanding of the differences between conscious attention and what is revealed with gaze data, I was surprised to see in my own results how many small attentional shifts and unconscious decisions I made while my conscious attention was focused somewhere else. My gaze behavior while teaching indicated an advanced level of situation awareness (23) that enabled me to make countless unconscious decisions that are driven by behavior patterns established through reinforcement learning over years of experience. These unconscious processes occur in all domains of expertise (24; 26), and they underscore why experts are not always effective at teaching their craft.

My conscious goal while teaching was to help the students produce a characteristic sound on the trumpet while performing Breathin’ Easy (20). I did not knowingly plan any specific sequences or rehearsal frames in advance, but my prior experience and long-term memory, specifically with these students, and with students generally, facilitated automatization of each micro-decision as it interacted with my plans to achieve my goals. Leading 9 performance trials on 3 different glissando patterns in 82 seconds, as I did in the first rehearsal frame, is impossible to execute and process in working memory without this type of expertise. These highly automatized and implicitly controlled components of teaching make it difficult for skillful teachers to articulate their thinking and challenging to teach novices to develop this kind of thinking.

Information integration and attention switching is a central yet unconscious component of my teaching: instead of spending long stretches of time attending only to Charles, who struggled with the performance tasks, I periodically switched my attention to Allen when there were strategic opportunities to do so (e.g., when Charles’ performance did not require my uninterrupted attention). A simple observation of this lesson, absent any gaze data, might also have concluded that I significantly prioritized Charles by giving him continuous feedback and setting the progression of the sequence to his playing level. My gaze behavior, not my memory, revealed how I divided my attention, even though many of my fixations on Allen were brief and not followed by overt actions. Monitoring and shaping student behavior are such rehearsed and reinforced tasks that the associated thought processes were completely automatized while I consciously monitored performance progress and made musical decisions. Ideal attention allocation strikes a balance to achieve the conscious and unconscious goals of the lesson: to help both students produce a characteristic sound on the trumpet and keep both students engaged in the lesson.

Although summative statistics showed broadly consistent gaze behavior within each condition, the nuances of my goal-directed attention became apparent when looking at small moments that were connected to student performance. When I examined my fixations and compared my gaze behavior to the context of the lesson, almost every fixation was clearly explainable and predictable based on student performance trials and my feedback and directives. I had conscious knowledge of my musical goal and how I needed to reach the goal, and I knew consciously when I accomplished the goal, but each small decision never rose to the level of conscious awareness while teaching. Annotating my rehearsal frames was the first time I noticed each attentional shift and each sequential step or proximal goal, even though I had done all these things in the moment. I had no idea how long I dwelled on each student, how often I switched my attention from one student to the other, how I searched for information about their playing, or when I decided whether to repeat a performance trial or move on to the next step. Even though I did not consciously control any of these actions, my gaze behavior reflects my thinking because all my fixations fit into the broad construal of my teaching goals.

Furthermore, my fixation patterns were quite different when I watched the video of my lesson. This study design allows for a high degree of control because my memories and expertise are present in both conditions, thus demonstrating clear relationships between active engagement in music teaching and its associated thought processes. Much like the difference between driving a car and being a passenger in a car, active teaching requires a great deal of thinking, predicting, and planning for what happens next. Passengers and observers, on the other hand, are free to pursue a variety of interesting or salient stimuli in the environment that may or may not be explicitly connected to achieving upcoming goals.

There are undoubtedly things to learn from observing teaching, but this study suggests that observations may have limitations, especially for preservice teachers, that have not been carefully considered. Overt activities like classroom observations may not reveal to novice teachers the unconscious processes that determine how teachers allocate attention moment to moment, especially considering that advantageous gaze behavior develops as a result of reinforcement learning. It is therefore worth pondering the role of observation in preservice teacher preparation programs and determining the types of experiences that are likely to contribute to novices’ skill development in teaching (27).

All of this illustrates that many noteworthy decisions happen below the surface of expert teachers’ conscious thinking and are inaccessible during active teaching, thus supporting the assertion that this research area is worth pursuing more. Further replication of this study design with more participants will provide further insight and generalizability about the unconscious components of expert music teachers’ thinking. Future studies should also analyze how teachers’ gaze varies when they attend to musical and behavioral goals; when they utilize top-down directed attention vs. looking for the source of an error; how they reference their musical score at the beginning, middle, end of rehearsal frames; how they monitor performance goals across multiple rehearsal frames; and other comparisons. Future studies should also explore the interaction between musical and behavioral goals by replicating this design with skillful teachers of younger students. Automaticity in behavior management skills may manifest differently in classroom settings with younger students, and this type of replication may uncover interesting interactions. Classroom music teachers spend their days in environments with more than two students, of course, and these fundamental questions of expertise and attention allocation must be investigated in more detail in constrained environments before meaningful insights can be explored in authentic contexts with more variability. The long-term goal for this line of research is to uncover the mechanisms that govern music teacher attention and situation awareness in large ensemble rehearsals with dozens of musicians and unlimited variability. Learning more about how expert teachers teach enables us to develop in others the genuine skills of expert teaching.

### Ethics and Conflict of Interest

The author declares that the contents of the article are in agreement with the ethics described in http://biblio.unibe.ch/portale/elibrary/BOP/jemr/ethics.html and that there is no conflict of interest regarding the publication of this paper.

### Acknowledgements

This research was supported in part by a grant from the Center for Music Learning in the Ernest and Sarah Butler School of Music at The University of Texas at Austin.
